# Clinical features and outcome of maintenance hemodialysis patients with COVID-19 from a tertiary nephrology care center in Romania

**DOI:** 10.1080/0886022X.2020.1853571

**Published:** 2020-12-13

**Authors:** Gabriel Stefan, Ana Maria Mehedinti, Iuliana Andreiana, Adrian Dorin Zugravu, Simona Cinca, Ruxandra Busuioc, Ioana Miler, Simona Stancu, Ligia Petrescu, Ioana Dimitriu, Elena Moldovanu, Diana Elena Crasnaru, Georgeta Gugonea, Valentin Georgescu, Victor Dan Strambu, Cristina Capusa

**Affiliations:** a “Dr. Carol Davila” Teaching Hospital of Nephrology, Bucharest, Romania; b Department of Nephrology, “Carol Davila” University of Medicine and Pharmacy, Bucharest, Romania; c Department of Surgery,“Carol Davila” University of Medicine and Pharmacy, Bucharest, Romania

**Keywords:** COVID-19, chronic hemodialysis, clinical features, mortality

## Abstract

**Background:**

There is limited information about the clinical characteristics, treatment and outcome of maintenance hemodialysis patients with COVID-19. Moreover, regional differences are also conceivable since the extend and severity of outbreaks varied among countries.

**Methods:**

In this retrospective, observational, single-center study, we analyzed the clinical course and outcomes of 37 maintenance hemodialysis patients (median age 64 years, 51% men) hospitalized with COVID-19 from 24 March to 22 May 2020 as confirmed by real-time PCR.

**Results:**

The most common symptoms at admission were fatigue (51%), fever (43%), dyspnea (38%) and cough (35%). There were 59% mild/moderate patients and 41% severe/critical patients. Patients in the severe/critical group had a significantly higher atherosclerotic burden since diabetic kidney disease and vascular nephropathies were the most common primary kidney diseases and eighty percent of them had coronary heart disease. Also, Charlson comorbidity score was higher in this group. At admission chest X-ray, 46% had ground-glass abnormalities. Overall, 60% patients received hydroxychloroquine, 22% lopinavir–ritonavir, 11% tocilizumab, 24% systemic glucocorticoids, and 54% received prophylactic anticoagulation. Seven (19%) patients died during hospitalization and 30 were discharged. The main causes of death were cardiovascular (5 patients) and respiratory distress syndrome (2 patients). In Cox regression analysis, lower oxygen saturation, anemia and hypoalbuminemia at admission were associated with increased mortality.

**Conclusions:**

In conclusion, we observed a high mortality rate among maintenance hemodialysis patients hospitalized for COVID-19. Anemia, lower serum albumin and lower basal oxygen saturation at admission were factors associated with poor prognosis.

## Introduction

Severe acute respiratory syndrome coronavirus 2 (SARS-CoV-2) infection emerged in Wuhan, China in December 2019 and rapidly developed in a pandemic. Since then, over 38 million infected cases with SARS-CoV-2 were globally reported [1].

In the general population, the 2019 coronavirus disease (COVID-19) has a mortality rate of around 6% [1], similar to SARS-CoV (10%) but lower than MERS-CoV (around 40%) [2–4].

Patients with comorbid conditions like obesity, hypertension, diabetes mellitus, cardiovascular disease, advanced age, or superimposed acute kidney injury were found to be more susceptible to SARS-CoV-2 infection, and to have higher risk of intensive care admission or death due to COVID-19 [4–10].

However, these findings were mainly obtained from studies on the general population.

Patients on maintenance hemodialysis appear particularly vulnerable to SARS-CoV-2 infection due to uremia-related immune system dysfunction which consist in both impaired immune defense and pro-inflammatory state, increased comorbidity burden, frequent hospital admissions and the risk of cross-contamination in the dialysis centers [10–12].

Most studies that included dialysis patients focused on infection susceptibility and strategies to limit the disease spread [11,13]. To date, only isolated observations and small case series on prevalence and mortality rate have been reported in this population [10,11,14–17]. Given the increased vulnerability of these patients, clinical presentation and outcome could be different from the general population. Moreover, regional differences are also conceivable since the extend and severity of outbreaks varied among countries.

Therefore, in this observational retrospective cohort study we aimed to assess the clinical presentation, treatment and outcome in hemodialysis patients with SARS-CoV-2 infection from a tertiary Nephrology care center in South-East Europe.

## Methods

All adult patients who were on maintenance hemodialysis therapy, and were admitted to our hospital with positive real-time reverse transcription-polymerase chain reaction (rRT-PCR) testing for SARS-CoV-2 between 24 March 2020, and 22 May 2020, were identified and enrolled in this retrospective observational study. The subjects were followed from the moment of admission to death or discharge.

The study protocol was approved by the Local Ethics Committee (No 14/April 2020 “Dr. Carol Davila” Teaching Hospital of Nephrology Ethics Committee).

Demographic, clinical data, radiological evaluation and laboratory tests (hemoglobin, white blood cells and platelets count, C-reactive protein, serum albumin, lactate dehydrogenase and liver function tests) at admission time were retrieved form patients’ files.

The primary kidney disease diagnosis was summarized in six categories: glomerular nephropathies, diabetic kidney disease, vascular nephropathies, tubulointerstitial nephropathies and polycystic kidney disease.

The degree of severity of COVID-19 was defined as mild, moderate, severe and critical as previously described [11]. Mild refers to patients who had mild clinical symptoms without manifestation of viral pneumonia on chest X-ray. Moderate refers to patients who had symptoms with manifestation of viral pneumonia on chest X-ray. Severe refers to adults who met any of the following criteria: (1) respiratory rate ≥30 breaths/min; (2) oxygen saturation ≤93% at rest state; and (3) arterial PO2/oxygen concentration ≤300 mmHg.

Patients with pulmonary lesion progression >50% within 24–48 h on radiologic imaging were treated as severe cases. Critical refers to patients that met any of the following criteria: (1) occurrence of respiratory failure requiring mechanical ventilation; (2) presence of shock; and (3) other organ failure that requires monitoring and treatment in the intensive care unit [11]. The patients were classified in two groups as mild/moderate and severe/critical; comparisons between the two groups were performed.

Acute Respiratory Distress Syndrome (ARDS) was defined according to the Berlin definition [18]. Cardiac injury was defined as the serum levels of cardiac biomarkers (e.g., troponin I) being above the 99th percentile upper reference limit, or if new abnormalities were detected via electrocardiography and echocardiography.

Treatment measures included: immunomodulatory, antiviral or antibiotic therapy, corticosteroid therapy, respiratory support, continuous renal replacement therapy. Patients with moderate, severe and critical disease received hydroxychloroquine; while severe and critical patients were treated with lopinavir–ritonavir; tocilizumab was indicated only for critical patients. However, the treatment was tailored according to patients comorbidities and clinical care needs. Also, the dialysis prescription was individualized according to previous regimes and clinical evolution.

Continuous variables are presented as mean or median and interquartile range (IQR), according to their distribution, and categorical variables as percentages. Group comparisons were performed with *t* test, Mann–Whitney *U* test and *χ*^2^ test, as appropriate.

The analysis of the variables related to survival was carried out using the multivariate Cox proportional hazard models. Adjustments were made for age and basal oxygen saturation.

A *p* value of 0.05 or less was considered statistically significant. All statistical analyses were performed with SPSS 21.0 software (Chicago, IL).

## Results

A total of 37 patients were included in the study. Eighty-five percent of the patients came from the same outpatient maintenance hemodialysis center.

The detailed baseline characteristics of the study population are displayed in Table 1. The median age of the study population was 64 (IQR 55–71) years, and 51% were male. Approximately one quarter of the patients were obese, and only 14% were current smokers.

**Table 1.** Characteristics of COVID-19 patients according to the severity of disease.

Characteristics	Total *N* = 37	Mild/moderate *n* = 22	Severe/critical *n* = 15	*p*
Age, years	64 (55–71)	62 (52–67)	67 (60–72)	0.1
Male, *n* (%)	19 (51)	14 (64)	5 (33)	0.07
Current smoker, *n* (%)	5 (14)	1 (5)	4 (27)	0.05
Primary cause of ESKD, *n* (%)				<0.01
Diabetic kidney disease	12 (32)	6 (27)	6 (40)	
Vascular nephropathy	7 (19)	1 (5)	6 (40)	
Glomerular nephropathy	4 (11)	4 (18)	0 (0)	
Polycystic kidney disease	5 (14)	2 (9)	3 (20)	
Tubulointerstitial nephropathies	6 (16)	6 (27)	0 (0)	
NA	3 (8)	3 (14)	0 (0)	
Comorbidities				
Coexisting disorder, *n* (%)				
Arterial hypertension	30 (81)	17 (77)	13 (87)	0.4
Coronary heart disease	19 (51)	7 (32)	12 (80)	<0.01
Diabetes mellitus	13 (35)	7 (32)	6 (40)	0.6
Chronic obstructive pulmonary disease	3 (8)	0 (0)	3 (20)	0.02
Atrial fibrillation	10 (27)	4 (18)	6 (40)	0.1
Cancer	2 (5)	1 (5)	1 (7)	0.7
Obesity, *n* (%)	11 (30)	5 (23)	6 (40)	0.2
Viral status				0.1
Hepatitis B virus	1 (3)	0 (0)	1 (7)	
Hepatitis C virus	4 (11)	1 (5)	3 (20)	
Charlson comorbidity index	7 (4–8)	5 (3–7)	8 (7–10)	<0.01
Previous medication, *n* (%)				
ACEI/ARB	3 (8)	0 (0)	3 (20)	0.02
Immunosuppressant	4 (11)	4 (18)	0 (0)	0.08
Hemodialysis data				
Hemodialysis vintage, months	38 (10–63)	23 (8–70)	45 (34–61)	0.2
Hemodialysis access, *n* (%)				0.4
Arteriovenous fistula	20 (54)	13 (59)	7 (47)	
Central venous catheter	17 (46)	9 (41)	8 (53)	
Clinical presentation and laboratory findings				
Symptoms, *n* (%)				
Fever	16 (43)	7 (32)	9 (60)	0.08
Cough	13 (35)	4 (18)	9 (60)	<0.01
Fatigue	19 (51)	8 (36)	11 (73)	0.02
Diarrhea	3 (8)	1 (5)	2 (13)	0.3
Nausea/vomiting	3 (8)	2 (9)	1 (7)	0.7
Dyspnea	14 (38)	2 (9)	12 (80)	<0.001
Sore throat	8 (22)	4 (18)	4 (27)	0.5
Basal oxygen saturation, %	95 (89–96)	96 (95–97)	88 (80–90)	<0.001
Admission chest X-ray, *n* (%)				<0.001
Bilateral ground glass opacity	12 (32)	0 (0)	12 (80)	
Unilateral opacity	5 (14)	2 (9)	3 (20)	
Normal X-ray	20 (54)	20 (91)	0 (0)	
Laboratory findings				
Hemoglobin, g/L	11.0 (9.9–11.9)	11.8 (10.4–12.2)	9.6 (7.5–11.0)	<0.001
Platelet, 10^9^/L	221 (158–301)	234 (179–308)	208 (137–301)	0.1
White blood cells, 10^9^/L	7.5 (5.3–9.9)	7.5 (5.3–8.9)	8.0 (5.0–14.2)	0.3
Lymphocytes, 10^9^/L	1.5 (1.2–2.1)	1.6 (1.2–2.2)	1.4 (1.0–2.1)	0.5
Neutrophils, 10^9^/L	4.9 (3.2–7.2)	4.8 (2.9–6.4)	5.4 (3.3–12.0)	0.1
C-reactive protein, mg/L	96 (23–192)	54 (5–164)	135 (41–198)	0.1
Serum albumin, g/dL	3.5 (3.0–3.9)	3.6 (3.2–4.3)	3.3 (2.8–3.7)	0.06
LDH, U/L	295 (227–424)	274 (204–332)	317 (251–473)	0.1
GOT, U/L	25 (18–37)	22 (13–37)	26 (19–37)	0.3
GPT, U/L	15 (12–30)	15 (12–27)	16 (10–32)	0.8
GGT, U/L	43 (19–66)	34 (20–57)	51 (19–69)	0.4
Complications, *n* (%)				<0.01
Cardiac injury	3 (8)	0 (0)	3 (20)	
Liver dysfunction	1 (3)	0 (0)	1 (7)	
Cerebrovascular event	1 (3)	0 (0)	1 (7)	
Acute respiratory distress syndrome	4 (11)	0 (0)	4 (27)	
Treatment and outcome				
Drugs, *n* (%)				
Lopinavir–ritonavir	8 (22)	3 (14)	5 (33)	0.1
Tocilizumab	4 (11)	0 (0)	4 (27)	0.01
Hydroxychloroquine	22 (60)	9 (41)	13 (87)	<0.01
Glucocorticoids	9 (24)	1 (5)	8 (53)	0.001
Anticoagulation	20 (54)	7 (32)	13 (87)	0.001
Antibiotics	18 (49)	7 (32)	11 (73)	0.01
CRRT, *n* (%)	5 (14)	0 (0)	5 (33)	<0.01
Mechanical ventilation, *n* (%)				
Noninvasive	2 (5)	0 (0)	2 (13)	0.07
Invasive	5 (14)	0 (0)	5 (33)	<0.01
Mortality, *n* (%)	7 (19)	1 (5)	6 (40)	<0.01

ACEI/ARB: angiotensin converting enzyme inhibitor/angiotensin receptor blocker; CRRT: continuous renal replacement therapy; ESKD: end-stage kidney disease; GGT: gamma-glutamyl transferase; LDH: lactate dehydrogenase; GOT: glutamic oxaloacetic transaminase; GPT: glutamic pyruvic transaminase; NA: not assessed.

The most common primary kidney diseases were diabetic kidney disease (32%) and vascular nephropathies (19%), followed by tubulointerstitial nephropathies (16%), polycystic kidney disease (14%) and glomerular nephropathies (11%). In our population, 95% had at least one comorbidity, arterial hypertension being the most frequent (81%), followed by coronary heart disease (51%), diabetes mellitus (35%) and atrial fibrillation (27%). Moreover, the median Charlson comorbidity index was seven. Other coexisting conditions like chronic obstructive pulmonary disease (8%), hepatitis B virus infection (3%), hepatitis C virus infection (11%) and cancer (5%) were rare (Table 1).

All patients underwent dialysis three times per week before the pandemic and the median dialysis vintage was 38 (IQR 10–63) months. More than half of the patients (54%) used arteriovenous fistula as hemodialysis access at admission time, and 46% of them used cuffed tunneled central venous catheter.

The most common symptoms at admission were fatigue (51%), fever (43%), dyspnea (38%) and cough (35%). There were 22 (59%) mild/moderate patients and 15 (41%) severe/critical patients. Patients in the severe/critical group had a significantly higher atherosclerotic burden since diabetic kidney disease and vascular nephropathies were the most common primary kidney diseases and eighty percent of them had coronary heart disease. Moreover, the median Charlson comorbidity score was higher in this group (Table 1).

Symptoms like fatigue, cough and dyspnea were more frequent in the severe/critical group. All complications, including ARDS, cardiac injury, cerebrovascular event and liver dysfunction, were found in the severe/critical group. With the exception of a higher grade of anemia in the severe/critical group, there were no significant differences in hematology and biochemical tests (Table 1).

On the admission chest X-ray, we observed abnormalities in 17 patients (46%). The typical radiologic pattern, consisting of peripheral ground-glass opacities, was bilateral in 12 patients and unilateral in 5 patients. All patients from the severe/critical group had peripheral ground-glass opacities (Table 1).

Overall, 8 (22%) patients received lopinavir–ritonavir for antiviral therapy, 22 (60%) patients received hydroxychloroquine and 4 (11%) received tocilizumab. Systemic glucocorticoids were used in only 9 (24%) patients. More than half of the patients received prophylactic anticoagulation (Table 1).

Antibiotherapy was used in 18 (49%) of the studied patients. Bacterial superinfection was found in 9 (24%) patients. *Enterecoccus sp* (4 patients), *Pseudomonas sp* (3 patients) and *Klebisella sp* (3 patients) were the most frequent isolated, followed by *Acinetobacter sp* (2 patients) and *E coli* (2 patients).

Tocilizumab was administered only in the severe/critical patients; also, these patients received more often treatment with hydroxychloroquine, systemic glucocorticoids, anticoagulation and antibiotics. Furthermore, noninvasive ventilation and mechanical ventilation were restricted to these patients (2 and 5 patients, respectively). Continuous renal replacement therapy was used only in the severe/critical group (5 patients), hemodynamic instability and overhydration being the major indications (Table 1). The rest of the patients received conventional high flux hemodialysis without schedule changes.

Seven (19%) patients died during hospitalization and 30 were discharged. The main causes of death were cardiovascular (5 patients) and respiratory distress syndrome (2 patients). Mortality was significantly higher in the severe/critical group (Table 1).

Positive history of coronary heart disease was present in all patients who died but one. Also, they had dyspnea at admission more frequently, lower oxygen saturation, higher grade of anemia and increased inflammation. Regarding the treatment, more than half of the patients from the non-survivors group received invasive mechanical ventilation and continuous renal replacement therapy. Furthermore, they received more often treatment with lopinavir–ritonavir, tocilizumab, systemic glucocorticoids and prophylactic anticoagulation (Table 2).

**Table 2.** Characteristics of COVID-19 patients according to survival.

Characteristics	Survivors	Non-survivors	*p*
	*n* = 30	*n* = 7	
Age, years	63 (55–68)	69 (55–72)	0.4
Male, *n* (%)	16 (53)	3 (43)	0.6
Current smoker, *n* (%)	3 (10)	2 (29)	0.1
Primary cause of ESKD, *n* (%)			0.2
Diabetic kidney disease	10 (34)	2 (29)	
Vascular nephropathy	4 (13)	3 (42)	
Glomerular nephropathy	4 (13)	0 (0)	
Polycystic kidney disease	3 (10)	2 (29)	
Tubulointerstitial disease	6 (20)	0 (0)	
NA	3 (10)	0 (0)	
Comorbidities			
Coexisting disorder, *n* (%)			
Arterial hypertension	25 (83)	5 (71)	0.4
Coronary heart disease	13 (43)	6 (86)	0.04
Diabetes mellitus	11 (37)	2 (29)	0.6
Chronic obstructive pulmonary disease	1 (3)	2 (29)	0.02
Atrial fibrillation	6 (20)	4 (57)	0.04
Cancer	1 (3)	1 (14)	0.2
Obesity, *n* (%)	8 (27)	3 (43)	0.3
Viral status			0.02
Hepatitis B virus	0 (0)	1 (14)	
Hepatitis C virus	2 (7)	2 (29)	
Charlson comorbidity index	7 (4–8)	9 (6–9)	0.1
Previous medication, *n* (%)			
ACEI/ARB	2 (7)	1 (14)	0.5
Immunosuppressant	4 (13)	0 (0)	0.3
Hemodialysis data			
Hemodialysis vintage, months	35 (8–70)	43 (22–58)	0.8
Hemodialysis access, *n* (%)			0.1
Arteriovenous fistula	18 (60)	2 (29)	
Central venous catheter	12 (40)	5 (71)	
Clinical presentation and laboratory findings			
Symptoms, *n* (%)			
Fever	12 (40)	4 (57)	0.4
Cough	9 (30)	4 (57)	0.1
Fatigue	14 (47)	5 (71)	0.2
Diarrhea	2 (7)	1 (14)	0.5
Nausea/vomiting	3 (10)	0 (0)	0.3
Dyspnea	8 (27)	6 (86)	<0.01
Sore throat	6 (20)	2 (29)	0.6
Basal oxygen saturation, %	95 (90–97)	88 (80–90)	<0.01
Admission chest X-ray, *n* (%)			<0.01
Bilateral ground glass opacity	7 (23)	5 (71)	
Unilateral opacity	3 (10)	2 (29)	
Normal X-ray	20 (67)	0 (0)	
Laboratory findings			
Hemoglobin, g/L	11.4 (10.3–11.9)	8.7 (7.4–11)	0.04
Platelet, 10^9^/L	223 (179–282)	216 (101–365)	0.8
White blood cells, 10^9^/L	7.3 (5.3–8.5)	13.8 (5.0–15.6)	0.07
Lymphocytes, 10^9^/L	1.7 (1.2–2.2)	1.1 (0.9–2.0)	0.1
Neutrophils, 10^9^/L	4.8 (3.2–7.1)	9.8 (3.3–14.1)	0.06
C-reactive protein, mg/L	67 (7–198)	135 (96–192)	0.3
Serum albumin, g/dL	3.7 (3.3–4.0)	2.8 (2.4–3.0)	0.001
LDH, U/L	291 (250–332)	424 (202–500)	0.6
GOT, U/L	24 (17–35)	36 (19–44)	0.2
GPT, U/L	16 (12–27)	13 (8–34)	0.5
GGT, U/L	43 (20–61)	51 (15–69)	0.9
Complications, *n* (%)			<0.01
Cardiac injury	0 (0)	3 (43)	
Liver dysfunction	1 (3)	0 (0)	
Cerebrovascular event	1 (3)	0 (0)	
ARDS	3 (10)	1 (14)	
Treatment			
Drugs, *n* (%)			
Lopinavir–ritonavir	3 (10)	5 (71)	<0.01
Tocilizumab	1 (3)	3 (43)	<0.01
Hydroxychloroquine	17 (57)	5 (71)	0.4
Glucocorticoids	3 (10)	6 (86)	<0.001
Anticoagulation	13 (43)	7 (100)	<0.01
Antibiotics	13 (43)	5 (71)	0.1
CRRT, *n* (%)	1 (3)	4 (57)	<0.001
Mechanical ventilation, *n* (%)			
Noninvasive	1 (3)	1 (14)	0.2
Invasive	1 (3)	4 (57)	<0.001

ACEI/ARB: angiotensin converting enzyme inhibitor/angiotensin receptor blocker; CRRT: continuous renal replacement therapy; ESKD: end-stage kidney disease; GGT: gamma-glutamyl transferase; LDH: lactate dehydrogenase; GOT: glutamic oxaloacetic transaminase; GPT: glutamic pyruvic transaminase; NA: not assessed.

The median time until discharge was 18 (IQR 15–28) days after symptom onset and 16 (IQR 12–25) days after admission, and the median time to death was 17 (IQR 12–28) days after symptom onset and 14 (IQR 11–21) days after admission.

Lower oxygen saturation at admission, anemia and hypoalbuminemia were associated with increased mortality. Treatment with lopinavir–ritonavir, tocilizumab and glucocorticoids was related to higher in-hospital mortality, but not when was adjusted for age and basal oxygen saturation. However, patients who received hydoxychloroquine had better in-hospital adjusted survival (Table 3).

**Table 3.** Risk factors associated with in-hospital death.

	Univariable HR (95% CI)	*p*	Adjusted HR^a^ (95% CI)	*p*
Age, years	0.97 (0.90–1.05)	0.5	0.93 (0.83–1.04)	0.2
Hemodialysis vintage, months	0.99 (0.98–1.01)	0.8	1.00 (0.97–1.02)	0.7
Obesity, yes	0.56 (0.11–2.82)	0.4	1.38 (0.25–7.58)	0.7
Current smoker, yes	0.13 (0.02–0.84)	0.03	4.48 (0.54–36.92)	0.1
Diabetes mellitus, yes	3.55 (0.41–30.41)	0.2	0.63 (0.06–6.76)	0.7
Coronary heart disease, yes	0.33 (0.03–2.97)	0.3	0.98 (0.05–17.54)	0.9
Charlson comorbidity index	1.08 (0.76–1.53)	0.6	0.84 (0.51–1.40)	0.5
Basal oxygen saturation, %	0.82 (0.71–0.94)	<0.01	0.79 (0.66–0.94)	<0.01
Hemoglobin, g/L	0.51 (0.32–0.83)	<0.01	0.49 (0.26–0.94)	0.03
Lymphocytes, 10^9^/L	0.46 (0.11–1.87)	0.2	0.49 (0.17–1.43)	0.1
C–reactive protein, mg/L	1.00 (0.99–1.01)	0.5	1.01 (0.99–1.02)	0.1
Serum albumin, g/dL	0.21 (0.05–0.82)	0.02	0.13 (0.02–0.82)	0.03
LDH, U/L	1.00 (0.99–1.00)	0.8	1.00 (0.99–1.00)	0.4
Lopinavir–ritonavir, yes	0.13 (0.02–0.76)	0.02	2.79 (0.41–18.87)	0.2
Tocilizumab, yes	0.11 (0.02–0.56)	<0.01	4.22 (0.73–24.42)	0.1
Hydroxychloroquine, yes	1.15 (0.18–7.11)	0.8	26.7 (1.15–61.9)	0.04
Glucocorticoids, yes	0.07 (0.00–0.63)	0.01	4.29 (0.29–62.67)	0.2

HR: hazard ratio; LDH: lactate dehydrogenase.

^a^ Age and basal oxygen saturation.

## Discussion

To date, there are few reports regarding COVID-19 impact on patients undergoing maintenance hemodialysis [14,19,20]. To the best of our knowledge, this is the first study from South-East Europe to assess the clinical characteristics, treatment and outcome of acute SARS-CoV-2 infection in this group of patients.

Depending on geographical region and the studied population, i.e., hospitalized versus outpatients, general population versus patients on hemodialysis, different clinical findings have been described.

Fever is the most common symptom noted in general population, irrespective of geographical region (99% in China [21], 94% in United States [22], 56% in Italy [23]). In our cohort of hemodialysis patients, fever was found less frequent (43%), similar to data reported by Yiqiong et al. [24]. This could be due to a lower inflammatory response, since hemodialysis patients with COVID-19 had decreased number of lymphocytes and lower serum level of inflammatory cytokines than in the general population [24].

We noted fatigue as the most frequent complaint (51%), in contrast with data from hemodialysis patients from Spain (25%) [14] and China (8%) [24]. More than half of patients with fatigue were in the severe/critical group who were more anemic, which might explain these differences.

In our patients, dyspnea and cough were also common symptoms (38% and 35%, respectively), less frequent than in general population (60.4% and 41.1%, respectively) [23], but comparable with data from European (40% and 44%, respectively) [14] and Asian hemodialysis patients (26% and 37.4%, respectively) [11]. However, lower rates of gastrointestinal manifestations (diarrhea, vomiting) than in hemodialysis patients from the Spanish cohort (8% versus 17%) were noticed [14]. Although in studies from general population, smell and taste changes were reported ranging from 34 to 87% [23,25], none of our patients or from previous studies on hemodialysis patients [10,11,14] had any anomaly in this regard.

Admission chest X-rays showed normal aspect in 54% of patients, while all the patients from the severe/critical group had typical radiologic pattern of peripheral ground-glass opacities. This is consistent with previous chest X-ray data from hemodialysis patients [14], but in contrast with reported chest CT results where all of the hemodialysis patients had radiologic changes [24].

Since clinical presentation in COVID-19 hemodialysis patients seems to be milder than in the general population, but with positive radiologic changes, CT scans could improve the diagnostic rate and management of these patients.

In the current cohort, lower oxygen saturation at admission was associated with mortality, in opposite with data reported by Goicoechea et al. [14], where this association was lost after age adjustment. However, this result could be related to the higher prevalence of chronic obstructive pulmonary disease and coronary heart disease in our non-survivors group.

Regarding laboratory features associated with in-hospital mortality, we found no relationship with previous described risk factors such as lymphopenia, elevated lactate dehydrogenase, increased C-reactive protein [26]. Nonetheless, we found two baseline parameters related to mortality: hypoalbuminemia and anemia. The impact on mortality of hypoalbuminemia in hospitalized SARS-CoV-2 patients has been previously reported in the general population [27]. In our end-stage kidney disease patients, low serum albumin could be related to the malnutrition-inflammation complex syndrome, which is an important risk factor for cardiovascular mortality [28]. Furthermore, anemia could also be related to the increased cardiovascular mortality in our cohort. However, this relationship was not reported in similar Chinese hemodialysis COVID-19 studies [15,29].

Zou et al. found that fever, dyspnea, and elevated d-dimer were independent risk factors for death in hemodialysis patients with COVID-19 [29]. Similarly, we found that the patients in the non-survivor group had dyspnea more frequently; however, there were no differences regarding the fever in our population.

Currently, the pharmacological approach to treating SARS-CoV-2 infection is considered to be a two-step approach [17]. In the first step, antiviral drugs such as chloroquine or hydroxychloroquine, lopinavir–ritonavir, remdesivir – are used due to their alleged inhibitory effect on viral entry and replication. In the second step, which typically begins after 7–10 days from the onset of symptoms, immunosuppressive and immunomodulatory drugs are thought to be of benefit because of the hyperinflammatory and cytokine release syndromes [30].

So far, there are no randomized controlled trials on therapies for COVID-19 patients who are on maintenance hemodialysis. Goicoechea et al. in a retrospective observational study on 36 hemodialysis patients reported a possible beneficial effect of corticosteroids and azithromycin treatment [14]. Of note, all patients but one from this study received hydroxychloroquine [14].

In our study, patients who received treatment with lopinavir–ritonavir, tocilizumab and glucocorticoids were much more likely to die. Nevertheless, the relationship was not apparent after adjustments for age and basal oxygen saturation. These results could be due to confounding by indication, i.e., sicker patients were more likely to be given these treatments.

Administration of hydroxychloroquine appeared to reduce the risk of death in our cohort. However, evidence on the benefits and harms of using hydroxychloroquine to treat COVID-19 is very weak and conflicting in the general population [31–33]. Therefore, these findings should be interpreted with caution owing to potential bias and residual confounding resulted from the observational design and small sample size of the study. End-stage kidney disease patients are unique in view of their immunosuppressed status and increased comorbidity index [17]. Thus, dedicated double-blinded randomized clinical trials should be conducted in this group of patients.

The mortality rate in our cohort of maintenance hemodialysis patients was similar to that reported by Yiqiong et al. (19% versus 16.2%) in China, but lower than that described in studies from Spain (30.5%) and Italy (25–41%) [14–16,34].

However, compared with the reports from general population, mortality was higher (19% versus 1.4–8%) and similar to that observed in the intensive care units (26%) [35,36]. These results may be explained by the older age of the patients and the increased comorbidity burden, notably the increased percentage of patients with positive history of coronary heart disease.

Recent autopsy data analyzing morphologic and molecular features of pulmonary specimens from patients who died from ARDS due to COVID-19 showed severe endothelial injury [37]. This suggest that the risk of developing severe form of COVID-19 is related to endothelial dysfunction. Due to the uremic milieu, hemodialysis patient has abnormal immune response and increased endothelial dysfunction which could increase the mortality risk [38,39].

Wu et al. conducted a retrospective, observational study on COVID 19 patients from Wuhan comparing the clinical presentation and outcome of 49 hemodialysis patients with 52 hospitalized patients without kidney failure [10]. They found a significantly higher rate of complications in patients on hemodialysis, including shock, acute respiratory distress syndrome, arrhythmia, and acute cardiac injury. Also, compared with controls, more patients on hemodialysis received noninvasive ventilation (25% versus 6%) and the mortality rate was higher (14% versus 4%) [10].

The main cause of death in our patients was cardiovascular followed by respiratory distress syndrome. These data are in line with a similar Wuhan cohort, but in contrast with the reports from Spain and Italy where respiratory distress syndrome was the principal cause of mortality [14–16].

Our study has a number of limitations. First, because of the small sample size from one single center, it was difficult to evaluate risk factors for mortality in regression models adjusted for more variables. Second, because of the retrospective nature of the study, laboratory tests like serum ferritin, procalcitonin, d-dimers were not assessed in all patients. Thus, their role as prognostic factors for in-hospital death was not evaluated. Third, due to the confounding by indication, the results related to treatment should be interpreted with caution.

In conclusion, we observed a high mortality rate among maintenance hemodialysis patients hospitalized for COVID-19. Anemia, lower serum albumin and lower basal oxygen saturation at admission were factors associated with poor prognosis.

## Data Availability

Datasets analyzed during the current study are available from corresponding author on reasonable request.

## Code availability

Available from the corresponding author on reasonable request.
